# Use of positron emission tomography in evaluation of brachial plexopathy in breast cancer patients

**DOI:** 10.1038/sj.bjc.6690074

**Published:** 1999-02

**Authors:** A Ahmad, S Barrington, M Maisey, R D Rubens

**Affiliations:** Clinical Oncology Unit, Guy's Hospital, Lambeth Palace Road, London, SE1 7EH, UK; Clinical PET Centre, St. Thomas's Hospital, Lambeth Palace Road, London, SE1 7EH, UK

**Keywords:** brachial plexopathy, positron emission tomography, computerized tomography

## Abstract

18-Fluoro-2-deoxyglucose (FDG) positron emission tomography (PET) has previously been used successfully to image primary and metastatic breast cancer. In this pilot study, 19 breast cancer patients with symptoms/signs referrable to the brachial plexus were evaluated with ^18^FDG-PET. In 11 cases computerized tomography (CT) scanning was also performed. Of the 19 patients referred for PET study, 14 had abnormal uptake of ^18^FDG in the region of the symptomatic plexus. Four patients had normal PET studies and one had increased FDG uptake in the chest wall that accounted for her axillary pain. CT scans were performed in 9 of the 14 patients who had positive brachial plexus PET studies; six of these were either normal or showed no clear evidence of recurrent disease, while three CTs demonstrated clear brachial plexus involvement. Of two of the four patients with normal PET studies, one has had complete resolution of symptoms untreated while the other was found to have cervical disc herniation on magnetic resonance imaging (MRI) scan. The remaining two patients almost certainly had radiation-induced plexopathy and had normal CT, MRI and PET study. These data suggest that ^18^FDG-PET scanning is a useful tool in evaluation of patients with suspected metastatic plexopathy, particularly if other imaging studies are normal. It may also be useful in distinguishing between radiation-induced and metastatic plexopathy. © 1999 Cancer Research Campaign


*British Journal of Cancer* (1999) **79**(3/4), 478–482
© 1999 Cancer Research Campaign
Article no. bjoc.1998.0074

### Use of positron emission tomography in evaluation of brachial plexopathy in breast cancer patients

**A Ahmad[1], S Barrington[2], M Maisey[2] and RD Rubens[1]**

[1]Clinical Oncology Unit, Guy's Hospital, and [2]Clinical PET Centre, St. Thomas's Hospital, Lambeth Palace Road, London SE1 7EH, UK

**Summary** 18-Fluoro-2-deoxyglucose (FDG) positron emission tomography (PET) has previously been used successfully to image primary and metastatic breast cancer. In this pilot study, 19 breast cancer patients with symptoms/signs referrable to the brachial plexus were evaluated with $^{18}$FDG-PET. In 11 cases computerized tomography (CT) scanning was also performed. Of the 19 patients referred for PET study, 14 had abnormal uptake of $^{18}$FDG in the region of the symptomatic plexus. Four patients had normal PET studies and one had increased FDG uptake in the chest wall that accounted for her axillary pain. CT scans were performed in 9 of the 14 patients who had positive brachial plexus PET studies; six of these were either normal or showed no clear evidence of recurrent disease, while three CTs demonstrated clear brachial plexus involvement. Of two of the four patients with normal PET studies, one has had complete resolution of symptoms untreated while the other was found to have cervical disc herniation on magnetic resonance imaging (MRI) scan. The remaining two patients almost certainly had radiation-induced plexopathy and had normal CT, MRI and PET study. These data suggest that $^{18}$FDG-PET scanning is a useful tool in evaluation of patients with suspected metastatic plexopathy, particularly if other imaging studies are normal. It may also be useful in distinguishing between radiation-induced and metastatic plexopathy.

**Keywords:** brachial plexopathy; positron emission tomography; computerized tomography

Brachial plexus neuropathy is a significant cause of pain and disability in breast cancer patients. The complicated anatomy of the plexus and its immediate anatomical relationship to blood and lymphatic vessels makes this a difficult area to image accurately. The two major causes of brachial plexopathy in breast cancer patients are metastatic invasion of and radiation damage to the plexus (Bagley et al, 1978; Cascino et al, 1983). By far the commonest is metastatic plexopathy, but differentiation between the two pathologies (often impossible clinically) is important to plan treatment optimally. Accurate imaging is also required to define evaluable disease for monitoring treatment efficacy (again not always possible clinically). Computerized tomography scanning has been used to assess the brachial plexus (Cooke et al, 1988; Moskovic et al, 1992), but it has several limitations, namely restriction to imaging in the axial plane and the neurovascular bundle of the axillary artery and brachial plexus may appear as a single structure when the administration of contrast medium is poorly timed (Rapaport et al, 1988). In a series of 46 patients with proven metastatic plexopathy, 11% of patients had normal CT scans (Cascino et al, 1983).

Positron emission tomography with $^{F}$18-fluoro-2-deoxy-D-glucose has previously been used successfully to detect a wide range of tumours, including brain, head and neck and lung carcinomas as well as lymphomas and soft-tissue sarcomas (Wahl et al, 1994; Okada et al, 1992; Strauss and Conti, 1991; DiChiro, 1986). The utility of PET in cancer imaging relies on the enhanced glycolytic rate of malignant cells compared with surrounding normal tissue, which results in increased uptake of the glucose analogue FDG into the cancer cells. $^{18}$FDG-PET has been used to image to all known sites of primary and metastatic breast cancer in a series of 12 patients and, in addition, revealed unsuspected nodal metastases (Wahl et al, 1991). Furthermore, PET imaging has been shown to have high specificity in the differentiation between benign and malignant breast tissue (Avril et al, 1996). To our knowledge, its use in imaging the brachial plexus has been reported only once, in three patients with suspected metastatic plexopathy. Two patients had normal scans and were reported to have had no symptom progression; the third patient (who had non-diagnostic CT and MRI imaging) had abnormal FDG uptake in the symptomatic brachial plexus, and subsequent surgical exploration confirmed metastatic plexopathy (Wald, 1994). We report here a pilot study using FDG-PET to image the brachial plexus in 19 breast cancer patients, of whom 17 had a suspected metastatic aetiology while two had suspected radiation plexopathy. Eleven patients had concurrent CT scans.

*Received 2 November 1996*
*Revised 1 July 1998*
*Accepted 9 July 1998*

*Correspondence to:* A Ahmad, Richard Dimbleby Department Cancer Research, Rayne Institute, St. Thomas's Hospital, Lambeth Palace Road, London SE1 7EH, UK

### PATIENTS AND METHODS

From August 1993 to September 1996, 19 breast cancer patients with symptoms/signs referrable to the brachial plexus were evaluated with $^{18}$FDG-PET. In 11 cases CT scanning was also performed. Only three patients had an MRI scan. In addition, three patients had follow-up FDG-PET studies after treatment with chemotherapy to correlate symptom responses with changes in imaging findings. Table 1 shows the clinical characteristics of the patients studied, including details of any previous anti-cancer treatment in the adjuvant or advanced setting. All of the patients who underwent photon beam irradiation to the plexus prior to symptoms of plexopathy received radiation doses less than or equal to 50 Gy (radiation doses and fractionation shown in Table

**Table 1** Clinical and imaging characteristics of patients in study

| Age | Stage at presentation | Previous treatment in adjuvant/advanced setting | Interval between diagnosis and plexopathy (years) | Concurrent disease at time of plexopathy | Symptoms/signs of plexopathy | CT scan | PET scan |
|---|---|---|---|---|---|---|---|
| 44 | II | Axillary sampling<br>Adjuvant DXT to breast/gland fields (45 Gy/15) | 11 | Nil | C6/7 sensory loss proximal/distal weakness | Normal | Normal<br>(MRI normal) |
| 63 | III | CT (EFC ×4), MRM and ax. cl.<br>Tamoxifen/prednisolone | 2 | Skeletal | Axilla/anterior chest wall pain | Normal | Increased FDG at left sternal edge |
| 57 | III | Simple mastectomy<br>DXT to breast/gland fields (46 Gy/23) | 9 | Lymphatic<br>Skeletal<br>Pulmonary | Pain, distal weakness | Minimal thickening | +ve |
| 57 | IV | Nil | Presented with plexopathy | Pleural<br>Lymphatic | Pain | – | +ve |
| 43 | I | E/b + ax. cl.<br>DXT to breast | 5 | Nil | Distal numbness/ weakness | – | Normal<br>(MRI: C6/7 disc herniation) |
| 46 | II | Mastectomy/ax. cl.<br>CT (EFC × 6)<br>DXT scf/upper axillary chain<br>Tamoxifen | 1 | Nil | Proximal pain | – | Normal |
| 30 | II | E/b + DXT to breast/gland fields<br>MRM (same side) + ax. cl. 4 years later<br>Adjuvant CMF and tamoxifen | 6 | Nil | Pain, depressed biceps/triceps reflexes | Minimal thickening | +ve (follow-up PET after doxorubicin normal)<br>Good symptom response |
| 62 | I | Wide excision and ax. cl., DXT to breast<br>Tamoxifen | 3 | Pulmonary<br>Lymphatic | Pain, all reflexes depressed | – | +ve |
| 73 | II | E/b and ax. cl.<br>DXT to breast<br>Tamoxifen<br>DXT for R scf recurrence (2 years after diagnosis) | 3 | Lymphatic<br>proximal/distal weakness | C5–7 numbness | Axillary mass | +ve |
| 27 | I | E/b + R ax. cl.<br>L. axillary recurrence<br>L. ax. cl. and CT (CMF+EFC) | 8 | Breast mass | Pain, distal weakness | normal | +ve (follow-up PET unchanged after CMF) |
| 49 | I | Partial mastectomy and ax. cl., DXT to breast | 22 | Lymphatic (FNA+ve)<br>Pleural<br>Skeletal | Dysaesthesiae, distal weakness<br>Horners syndrome | – | +ve |
| 47 | III | CT (EFC × 4)<br>Mastectomy and ax. cl., tamoxifen | 2 | Lymphatic | Pain, distal weakness | Axillary mass | +ve |
| 36 | II | E/b and ax. cl., CT (CMF)<br>DXT to breast<br>Tamoxifen | 2 | Lymphatic | Pain,<br>sensory loss | Normal | +ve |
| 44 | I | E/b and DXT to breast and gland fields | 9 | Pulmonary<br>Lymphatic<br>Skeletal | Pain (axilla) | – | +ve (follow-up PET after EFC normal).<br>Good symptom response |
| 51 | I | MRM and ax. cl.<br>Tamoxifen | 3 | Anterior axillary fold mass (FNA +ve) | Distal sensory loss | Normal | +ve |
| 55 | II | Mastectomy and ax. cl. CT (CMF)<br>Tamoxifen | 4 | Lymphatic<br>Pulmonary | Pain, distal weakness | – | +ve |
| 48 | II | Simple mastectomy and axillary node biopsy<br>DXT axilla and scf (45 Gy/15) | 10 | Nil | Distal weakness, distal sensory loss, absent reflexes | – | Normal (MRI normal) |
| 42 | II | E/b and ax. cl.<br>CT (CMF ×6)<br>DXT breast | 2 | Lymphatic | Distal weakness, pain | Axillary mass | +ve |
| 50 | II | E/b and ax. cl.<br>CT (CMF ×6)<br>DXT breast<br>Tamoxifen | 3′ | Skeletal<br>Lymphatic | Pain, sensory loss | Minimal thickening | +ve |

CT, chemotherapy; DXT, radiotherapy; MRM, modified radical mastectomy; ax. cl., axillary clearance; E/b, excison biopsy; scf, supraclavicular fossa. CMF: cyclophosphamide (100 mg $m^{-2}$ orally days 1–14), methotrexate (40 mg $m^{-2}$ intravenously days 1 and 8) and 5-fluorouracil (600 mg $m^{-2}$ intravenously days 1 and 8). EFC: epirubicin (70 mg $m^{-2}$ intravenously every 3 weeks), cyclophosphamide (700 mg $m^{-2}$ intravenously every 3 weeks) and 5-fluorouracil (700 mg $m^{-2}$ intravenously every 3 weeks). Where indicated MRI was performed. Unless indicated otherwise, DXT was 50 Gy in 25 fractions to breast (and gland fields where indicated with midplane dose for axilla defined as 50 Gy).

*© Cancer Research Campaign 1999*

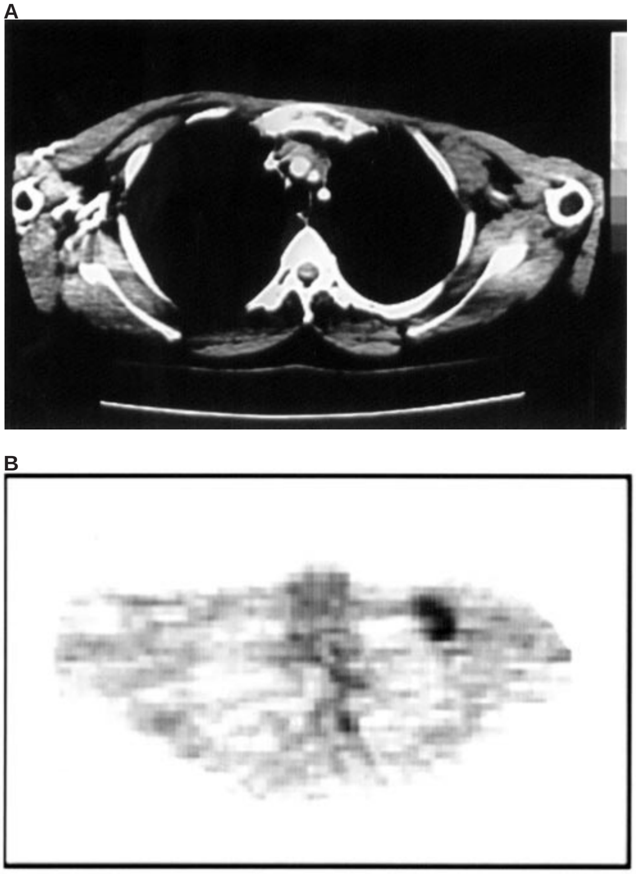

**Figure 1** (**A**) CT and (**B**) transaxial FDG-PET scan of a patient with symptoms and signs of a left brachial plexopathy. The CT scan shows a discrete left axillary mass that can be seen as an area of increased FDG uptake on the PET image

1). Only two patients received more than 2.5 Gy per fraction (both had radiation-induced brachial plexopathy). In all cases in which both chemotherapy and radiotherapy were administered in the adjuvant setting, chemotherapy was completed at least 3 weeks before irradiation was started.

Fluoride-18 was produced in a Siemens RDS cyclotron by proton bombardment of a high-pressure water target. FDG was synthesized by the method of nucleophilic substitution of a precursor by $^{18}$F. All PET scans were performed after a 6-h fast using an ECAT 951 body scanner (Siemens CTI, Knoxville, TN, USA). Thirty-one slices are produced over a 10.6-cm axial field of view. Transmission images were acquired initially using rotating Ge68 rod sources and were later used for attenuation correction in image reconstruction. Two adjacent emission images of 20 min duration each were acquired 45 min after injection of 250 MBq of FDG, commencing at the level of the clavicle to include the brachial plexus and axillae. The complete set of image planes were reconstructed by filtered back-projection using a Hann filter with a cut-off value of 0.4 × Nyquist frequency. A three-point smooth with 3.5 Gaussian width was applied in the axillary direction to obtain a single dataset with a final spatial resolution of 10 mm in all three orthogonal directions. CT scans were performed from C5 to the level of the arch of the aorta. Contiguous 5-mm slices were obtained using a Phillips SR7000 scanner with the patients' arms by their side. Intravenous contrast was not administered.

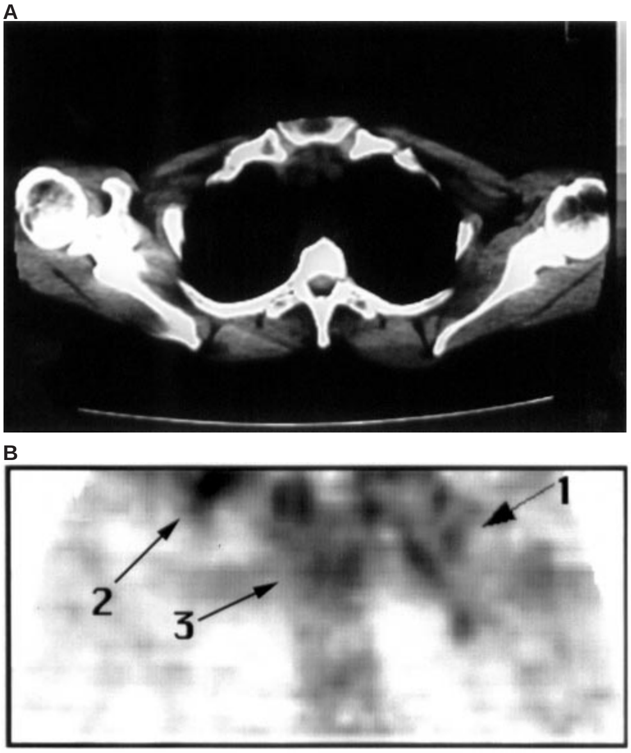

**Figure 2** (**A**) CT and (**B**) coronal FDG-PET scan from a patient with symptoms and signs of a left brachial plexopathy. CT scan of the left axilla shows no evidence of brachial plexus infiltration with preservation of the fat planes. Coronal PET image reveals marked FDG uptake along the left brachial plexus (arrow 1), both supraclavicular fossae (arrow 2 for right supraclavicular fossa) and the mediastinum (arrow 3)

### RESULTS

Of the 19 patients referred for PET study, 14 had abnormal uptake of $^{18}$FDG in the region of the symptomatic brachial plexus. Four patients had normal PET studies and one had increased FDG uptake at the left sternal edge, which accounted for her symptoms. CT scans were performed in 9 of the 14 patients who had positive brachial plexus PET studies; six of these were either normal or showed no clear evidence of recurrent disease, while three CTs demonstrated unequivocal brachial plexus involvement. These results are best interpreted when divided into the four following groups.

### 1 Positive PET and CT scans: three patients

All three CT scans revealed axillary masses. In one case there was clear confluent extension around the plexus, whereas in the other two there was no evidence of brachial plexus infiltration with preservation of the fat planes around the plexus. PET study showed clear infiltration *along* the plexus in two cases as well as sites of *focal* uptake in each axilla (all three cases), probably corresponding to the masses on CT scan (Figure 1). It is important to note that CT in one of the above cases also revealed multiple lung and mediastinal nodal metastases, which were confirmed as areas of increased FDG uptake on PET study.

### 2 Positive PET, negative CT scans: seven patients

Six of the seven patients had increased FDG uptake along the plexus on the symptomatic side; in three cases CT scanning

 © Cancer Research Campaign 1999

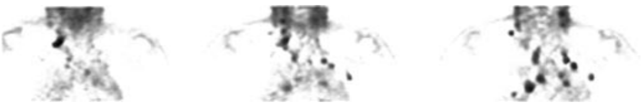

**Figure 3** Sequence of coronal PET images from a patient with symptoms and signs of a right brachial plexopathy showing uptake of FDG in the right brachial plexus, bilateral cervical lymph nodes, mediastinum, hila and left axilla revealed minimal increased thickness of the abnormal plexus in comparision with the other asymptomatic side. The remaining three CT scans were normal (Figure 2).

Three patients proceeded to radiotherapy; two had mild symptom response (less shooting pain/numbness), though both were also receiving concurrent analgesia; the third had no significant symptom response. The other three patients received palliative chemotherapy (CMF in two cases, doxorubicin in one case) – one had no symptom benefit (although there was objective evidence of response with a decrease in the size of an ipsilateral supraclavicular lymph node) – the remaining two patients had more significant symptom responses and repeat PET study in these cases revealed complete resolution of FDG brachial plexus uptake in one case and no change in the other.

The seventh patient in this group had complained of left chest wall and axillary pain, which was associated with weakness of her fingers during the episodes of pain. She had mild thumb abduction weakness on examination. CT scan of her left axilla and brachial plexus was normal. PET scan revealed increased FDG uptake at the left sternal edge adjacent to the attachment of the sixth rib but no evidence of brachial plexus uptake; palliative radiotherapy was administered with complete resolution of chest and axillary pain. There has been no progression of left arm symptoms for 2 years.

### 3 Positive PET, no CT scans five patients

All five patients had clinical evidence of either locoregional (supraclavicular fossa or axillary node) or distant recurrence (new pleural effusion) as well as brachial plexopathy. Three of the above received chemotherapy (one received a combination of epirubicin, cyclophosphamide and 5-fluorouracil, the other two received doxorubicin) with good subjective and objective response with follow-up FDG-PET in one case returning to normal. The fourth patient has more recently started chemotherapy (epirubicin) to treat plexopathy and distant metastatic disease; the fifth patient underwent local radiotherapy followed by systemic treatment with tamoxifen and experienced an initial improvement in hand strength as well as a response in her metastatic bone disease (fall of alkaline phosphatase to normal). Figure 3 shows serial coronal PET images from one of the above patients who developed symptoms and signs of a right brachial plexopathy.

### 4 Negative PET scans: four patients

The first patient complained of left upper arm pain 1 year after completing radiotherapy to her left supraclavicular fossa and upper axillary chain for local recurrence. There were no positive neurological signs and PET study was normal. Her symptoms have since completely resolved.

The second patient with a normal PET study had cervical disc herniation visible on MRI that accounted for her symptoms (MRI of brachial plexus was normal).

The third patient had a right simple mastectomy and low axillary clearance in 1979 for a 1.5 cm infiltrating intraductal carcinoma with one positive axillary lymph node. She then underwent post-operative radiotherapy with semi-opposed fields on a cobalt-60 teletherapy unit to a total tumour dose of 45 Gy in 15 fractions over 3 weeks. She remained well for 11 years, after which she developed numbness in the C6/7 dermatome. CT scanning and MRI revealed no evidence of recurrence. Over the next 4 years she developed progressive weakness of her right arm and hand with marked wasting of deltoid, pectoralis major and supraspinatus. Sensory signs remained confined to the lower dermatome and the triceps jerk was absent. Repeat MRI scan was normal, as was chest radiography, bone scan and abdominal ultrasonography. Based on the history, physical findings and lack of evidence of disease recurrence (locoregionally and distant sites) over 5 years, this patient almost certainly has radiation-induced brachial plexopathy. FDG-PET study in this case was also normal. The fourth patient in this group presented in 1979 with two lumps in the left breast (2.5 cm and 2 cm). Excision biopsy confirmed infiltrating carcinoma in both lesions extending into fat and perineural spaces. She proceeded to left simple mastectomy and axillary node biopsy (one axillary node contained metastatic carcinoma). Post-operatively she underwent irradiation to the left axilla and supraclavicular fossa with semiopposed fields on a cobalt-60 teletherapy unit (45 Gy in 15 fractions). She remained well for 10 years, when she developed paraesthesiae in her left hand. She had left carpal tunnel decompression in 1991 without any improvement. Numbness in the medial aspect of the left hand developed and electromyography (EMG) suggested a diffuse brachial plexus lesion. MRI scanning showed no evidence of recurrence and the brachial plexus appeared normal. There was, and never has been, any clinical or radiological (liver ultrasound, chest radiography and bone scan) evidence of disease recurrence elsewhere. Progression of plexopathy over recent years has resulted in marked distal weakness and wasting, loss of biceps/triceps/supinator reflexes and numbness in C8 dermatome. FDG-PET study in this case was also normal.

### DISCUSSION

This study demonstrates the feasibility of imaging the brachial plexus in cancer patients with intravenously injected $^{18}$FDG and a PET imaging device. CT has been used to study the brachial plexus (Cascino et al, 1983; Moskovic et al, 1992), but it is clear that it can be unreliable in this area. Data presented here show that six patients who had metastatic plexopathy (presence of concurrent locoregional disease or subsequent response to anti-cancer treatment) had either normal CT scans or the appearance of minimal thickening of the plexus. It is possible that this latter appearance may be related to previous axillary nodal dissection or radiotherapy. The three patients with positive CT scans had clearly delineated axillary masses, which is the commonest imaging finding in cases in which CT is helpful (Cascino et al, 1983; Thyagarajan et al, 1995). However, in one of the above cases, the appearance of the brachial plexus was normal, whereas FDG-PET showed clear uptake along the plexus. Furthermore, CT is not able to differentiate tumour infiltration from radiation fibrosis (Cascino et al, 1983). MRI has been claimed to be the test of choice in evaluating brachial plexopathy (Sherrier and Sostman, 1993). Its superiority to CT scanning relates to its multiplanar capability (axial, coronal and sagittal planes) and ability to differentiate more accurately nerves from surrounding vessels and soft tissues (Rapaport

© Cancer Research Campaign 1999

et al, 1988; Sherrier and Sostman, 1993). Review of the literature suggests that there is no doubt that MRI can delineate both the normal and abnormal anatomy of the brachial plexus in more detail than can CT scan (Ahern et al, 1991; Sherrier and Sostman, 1993; Panasci et al, 1995). Again, the presence of a discrete mass in relation to the plexus is the commonest finding in patients with metastatic plexopathy, although similar findings have been reported in radiation plexopathy patients (Thyagaragan et al, 1995). Indeed, in the largest published study to date on MRI imaging of brachial plexopathy in cancer patients, the overall MRI diagnosis was in disagreement with the clinicopathological diagnosis in 21% of patients (Thyagaragan et al, 1995). Regarding the distinction between radiation and neoplastic plexopathy using MRI, there are encouraging data that tumour recurrence has a higher signal intensity than radiation fibrosis on T2-weighted images (Glazer et al, 1985; Rapaport et al, 1988), although other authors have reported increased T2 signal in both metastatic and radiation plexopathy patients (Thyagaragan et al, 1995).

The results presented here suggest that FDG-PET is a useful imaging technique to study the brachial plexus in breast cancer patients (especially when CT has been unhelpful) and that it may be useful to monitor treatment efficacy (post-treatment scans in three cases revealed resolution of uptake in two and no change in the third). Indeed, PET has previously been reported to be of use in monitoring treatment responses in breast cancer patients (Huovinen et al, 1993; Wahl et al, 1993). Larger studies correlating changes in FDG-PET appearances of the brachial plexus with changes in clinical findings during and after treatment are required, however. The data here are insufficient to suggest unequivocally that FDG-PET may be useful in distinguishing neoplastic and radiation plexopathy as there were only two clear-cut cases of the latter. Of the four patients who had increased FDG uptake in axillae that had previously been treated with radiotherapy, two had good clinical and radiological responses to chemotherapy, a third patient had evidence of new and active locoregional disease and the fourth had an axillary mass visible on CT scan. In addition, one of the patients who responded to chemotherapy also had distant sites of FDG uptake that resolved post treatment. However, it is encouraging that, in the two patients whose most likely diagnosis was radiation-induced plexus damage, the FDG-PET study was normal, suggesting an aetiology distinct from carcinoma or inflammation. The underlying pathological process in radiation plexus damage is chronic fibrosis, and it would seem unlikely that significant increased FDG uptake will occur in such areas. Indeed, FDG-PET is being used to differentiate recurrent tumour from post-operative/radiotherapy scar tissue in the pelvis and brain (Patronas et al, 1982; DiChiro et al, 1988; Sigurdson and Cohen, 1991). If it is shown to be similarly reliable in the axilla then FDG-PET may be useful in resolving this diagnostic dilemma.

### CONCLUSION

$^{18}$FDG-PET scanning is a useful tool in the evaluation of patients with suspected metastatic plexopathy, particularly if other imaging studies are normal. As a result of its cost and limited availability at present, its routine use to investigate the brachial plexus cannot be recommended as yet until comparision is made with MRI, the investigation of choice at present, and correlated with clinical findings before and after treatment.

### REFERENCES

Ahern V, Soo YS and Langlands AD (1991) MRI scanning in brachial plexus neuropathy. *Australasian Radiol* **35**: 379–381

Avril N, Dose J, Janicke F, Ziegler S, Laubenbacher C, Romer W, Pache H, Herz M, Allgayer B, Nathrath W, Graeff H and Schwaiger M (1996) Metabolic characterisation of breast tumors with positron emission tomography using F-18 fluorodeoxyglucose. *J Clin Oncol* **14**: 1848–1857

Bagley FH, Walsh JW, Cady B, Salzman FA, Oberfield RA and Pazianos AG (1978) Carcinomatous versus radiation induced brachial plexus neuropathy in breast cancer. *Cancer* **41**: 2154–2157

Cascino TL, Kori S, Krol G and Foley KM (1983) CT of the brachial plexus in patients with cancer. *Neurology* **33**: 1553–1557

Cooke J, Cooke D and Parsons C (1988) Anatomy and pathology of the brachial plexus as demonstrated on computed tomography. *Clin Radiol* **39**: 595–601

Dichiro G (1986) Positron emission tomography using (18-F) fluorodeoxyglucose in brain tumours; a powerful diagnostic and prognostic tool. *Invest Radiol* **22**: 360–371

Dichiro G, Oldfield E, Wright DC, DeMichele D, Katz DA, Patronas NJ, Doppman JL, Larson SM, Ito M and Kufta CV (1988) Cerebral necrosis after radiotherapy and/or intraarterial chemotherapy for brain tumors: PET and neuropathological studies. *Am J Radiol* **150**: 189–197

Glazer HS, Lee JKT, Levitt RG, Heikin JP, Ling D, Totty WG, Balfe DM, Emani B, Wasserman TH and Murphy WA (1985) Radiation fibrosis: differentiation from recurrent tumor by MR imaging. *Radiology* **156**: 721–726

Huovinen R, Leskinen-Kallio S, Nagren K, Lehikoinen P, Ruotsalainen U and Teras M (1993) Carbon-11-methionine and PET in evaluation of treatment response of breast cancer. *Br J Cancer* **67**: 787–791

Moskovic E, Curtis S, A'hern RP, Harmer CL and Parsons C (1992) The role of diagnostic CT scanning of the brachial plexus and axilla in the follow-up of patients with breast cancer. *Clin Oncol* **4**: 74–77

Okada J, Yoshikawa K and Itami M (1992) Positron emission tomography using fluorine-18-fluorodeoxyglucose in malignant lymphoma: a comparision with proliferative activity. *J Nucl Med* **33**: 325–329

Panasci DJ, Holliday RA and Shpizner B (1995) Advanced imaging techniques of the brachial plexus. *Hand Clin* **11**: 545–553

Patronas NJ, DiChiro G, Brooks RA, DeLaPaz RA, Cornblith PL, Smith HH, Rizzoli HV, Kessler RM, Manning RG, Channing M, Wolf AP and O'Connor CM (1982) 18 fluordeoxyglucose and positron emission tomography in the evaluation of radiation necrosis of the brain. *Radiology* **144**: 885–889

Rapaport S, Blair DN, McCarthy SM and Desser TS (1988) Brachial plexus: correlation of MR imaging with CT and pathologic findings. *Radiology* **167**: 161–165

Sherrier R and Sostman HD (1993) MRI in traumatic and non-traumatic brachial plexopathy. *J Thor Imag* **8**: 27–33

Sigurdson ER and Cohen MC (1991) The applications of PET in clinical oncology. *J Nucl Med* **32**: 623–648

Strauss LG and Conti PS (1991) The applications of PET in clinical oncology. *J Nucl Med* **32**: 623–648

Thyagaragan D, Cascino T and Harms G (1995) Magnetic Resonance Imaging in brachial plexopathy of cancer. *Neurology* **45**: 421–427

Wahl RL, Cody RL and Hutchins GD (1991) Primary and metastatic breast carcinoma: initial evaluation with PET and the radiolabelled glucose analogue 18-fluorodeoxyglucose. *Radiology* **179**: 765–770

Wahl RL, Zadany K, Helvie M, Hutchins GD, Weber B and Cody R (1993) Metabolic monitoring of breast cancer chemohormonotherapy using positron emission tomography: initial evaluation. *J Clin Oncol* **11**: 2101–2111

Wahl RL, Quint LE and Greenough RL, Meyer CR, White RI and Orringer MB (1994) Staging of mediastinal non-small cell lung cancer with FDG-PET, CT and fusion images: preliminary prospective evaluation. *Radiology* **191**: 371–377

Wald JJ and Wahl RL (1994) Use of positron emission tomography in evaluation of brachial plexopathy. *Neurology* **44** (suppl. 2): 307A

 © Cancer Research Campaign 1999
